# Enabling data-driven discoveries in evolutionary genetics and genomics

**DOI:** 10.1093/genetics/iyaf084

**Published:** 2025-05-20

**Authors:** Sudhir Kumar

**Affiliations:** Institute for Genomics and Evolutionary Medicine, Temple University, Philadelphia, PA 19122, USA; Department of Biology, Temple University, Philadelphia, PA 19122, USA

**Keywords:** software, evolution, genomics, phylogenetics, databases, TimeTree, evolutionary patterns

## Abstract

The George W. Beadle Award honors individuals who have made outstanding contributions to the community of genetics researchers as a whole and led an exemplary research career. The 2025 awardee is Sudhir Kumar from Temple University, who has not only pushed the intellectual frontier of evolutionary genetics but has also served the community through numerous contributions to creating, disseminating, maintaining, and advancing invaluable software for molecular evolutionary genetics analyses (MEGA) and a web-accessible resource for species divergence times (TimeTree). In the essay below, Kumar traces the initiation and evolution of these resources and explains how these developments have driven his research program to develop computationally efficient and environmentally friendly innovations to address the growing need to analyze increasingly larger sequence data sets.
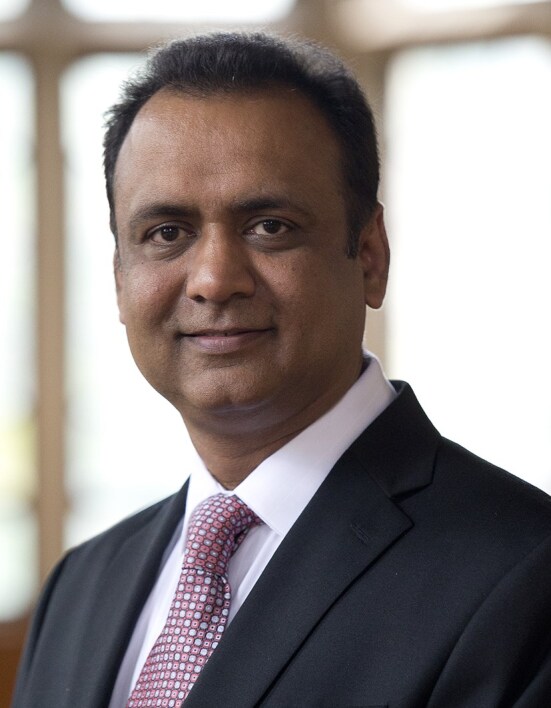

## A formative synthesis of biology and engineering

As an undergraduate pursuing dual degrees in Engineering and Biological Sciences, I learned to toggle comfortably between the conceptual worlds of algorithms and alleles. The engineering curriculum instilled a framework for problem-solving using mathematics and informatics, teaching the art of abstraction, optimization, and decomposition of complex problems. Biology courses sparked my curiosity about the genetic basis of the natural world. Consequently, I became fascinated with the intersection of computation and genetics, leading to a master's thesis on “Computer Simulation in Population Genetics” under the guidance of Prof. Sandhya Mitra at the Birla Institute of Technology and Science in India. At its conclusion, I was convinced that computation could serve as another instrument, like a microscope, in biology, which led me to pursue doctoral studies in molecular evolutionary genetics with Masatoshi Nei at Pennsylvania State University in the United States (Kumar 1996a).

The aim of my dissertation, data-driven discovery in evolutionary genetics through computational modeling and analysis, immediately encountered technological limits. While data sets were minuscule by today's standards, the computing power and tools for comparative sequence analysis were even less robust. Existing programs did not interact with one another and were not available for all the distance methods and algorithms that piqued my interest. Analyzing a data set inevitably involved rewriting code and translating file formats, often both. This bottleneck motivated me to create a single, integrated suite of C++ programs that implemented distance methods, particularly those pioneered by Masatoshi Nei's laboratory, where I trained. Another purpose for writing these programs from scratch was to learn various methods for computing evolutionary distances and reconstructing molecular phylogenies thoroughly through coding, which deepened my appreciation for their ingenuity, assumptions, and limitations.

## 1991–1993: MEGA 1 takes shape

What began as a personal toolbox soon evolved into the Molecular Evolutionary Genetics Analysis (MEGA) software. Masatoshi Nei's laboratory had a history of freely distributing computer programs for individual methods and algorithms, making it an ideal place for me to create comprehensive software that estimates various types of evolutionary distances and infers trees without requiring users to juggle multiple executables. However, MEGA's development hinged on 2 technical advances. First, most personal computers had limited memory and processing power in the early 1990s, necessitating numerous algorithmic and methodological innovations to minimize computer memory and time demands. Consequently, I developed new data structures and algorithms optimized for the average user's 640 kilobytes of available memory. I also wrote a program for generating UPGMA trees and devised new algorithms for reconstructing phylogenies using maximum parsimony optimality principle (Kumar *et al.* 1993). Both were popular in the early 1990s, and we deemed their inclusion necessary to provide a more complete set of calculation options for the user.

Second, a user-friendly interface was essential for conducting computational analyses because many biologists were unfamiliar with programming and command-line tools. Using Borland's TurboVision toolkit, I implemented a mouse-driven, text-based user interface, which included menus and dialog boxes, to execute my analysis code. The individual elements of MEGA's user interface evolved organically. For instance, the sequence data explorer was created to investigate multiple sequence alignments and calculate basic statistical properties of sequences (Kumar *et al.* 1993). Visual exploration of the results was further enhanced when postdoctoral fellow Koichiro Tamura translated his Modula-2 program for displaying NJ trees into C++. Meanwhile, Dr. Masatoshi Nei wrote explanations for various statistical methods and algorithms featured in the 140-page software manual (Kumar *et al.* 1993), which served as a precursor to our textbook, Molecular Evolution and Phylogenetics (Nei and Kumar 2000).

The result was a unique software package featuring a user-friendly interface, an extensive repertoire of evolutionary distance and maximum parsimony methods, and a detailed user manual suitable for a wide range of skill levels. A trial run in a graduate seminar taught by Masatoshi Nei in 1992 yielded many usability and programming insights, along with bug reports, that we used to enhance MEGA. MEGA version 1.0 (MEGA 1) officially debuted at the inaugural meeting of the Society for Molecular Biology and Evolution, organized by Walter Fitch. We decided to provide MEGA free of charge, despite some commercial interest, as we wanted everyone to benefit from advances in evolutionary methods (Kumar *et al.* 1994). This turned out to be a key decision in promoting the use of evolutionary genetics, as we received over 2,500 requests for MEGA 1 from around the world.

MEGA software version 1 was distributed by the postal mail, because the Internet was still in its infancy during the early 1990s. Each request meant mailing floppy diskettes (both 3½ and 5¼ inches) tucked safely inside padded envelopes, accompanied by a spiral-bound 140-page manual. At the time, I found myself moonlighting as an production manager, in addition to being a student pursuing a doctoral degree in Genetics. I oversaw contracts for mass-producing manuals and duplicating diskettes. Also, I printed and attached labels to all the diskettes mailed! This continued until I departed from Penn State in 1998.

## 1994–2001: discoveries and opportunities drive redesign—MEGA 2

During my graduate student years, I used MEGA 1 for data-driven discoveries, investigating the evolutionary trajectories of hedgehog and PAX multigene families and estimating species divergence times (Hedges *et al.* 1996; Kumar 1996b; Kumar *et al.* 1996; Balczarek *et al.* 1997; Kumar and Hedges 1998). These data sets continued to grow in both the number of sequences and their length, prompting the need to improve MEGA. Simultaneously, desktop computers gained in memory and speed, while Microsoft Windows offered enhanced high-resolution graphical user interfaces. The widespread adoption of MEGA 1 also led to the development of MEGA 2. I decided to undertake a complete rewrite of the MEGA source code to transition from a text-based to a graphical user interface and expand methodological offerings. I redesigned the user interface and rewrote every module using Borland's Delphi with the Object Pascal programming language. Koichiro Tamura wrote a new tree explorer. The result, MEGA 2 (Kumar *et al.* 2001), was launched via the newly minted website, www.megasoftware.net. Again, MEGA 2 was made freely available to all users, including researchers, students, and commercial users. Within the first year, MEGA 2 was downloaded more than 15,000 times by over 7,000 unique users from the website. At that time, MEGA was being cited more than 500 times a year, which is a testament to the growing popularity of molecular evolutionary approaches in biological research.

## 2002–2024: continuous innovation to meet challenges

Over the next 2 decades, MEGA has evolved in lockstep with advances in genomics, statistical methodologies, and the computing while adhering to our founding principles of producing user-friendly software for a wide range of sophisticated and efficient methods that meet the increasing needs of users. Milestones include the addition of an intuitive sequence alignment builder, the implementation of maximum likelihood and Bayesian approaches, the development of programs for estimating divergence times, and the creation of adaptive strategies to accelerate model selection and bootstrap analysis (Caspermeyer 2018; Kumar *et al.* 2024). Koichiro Tamura has led the implementation of many of these new calculations, becoming an equal partner in the evolution of MEGA. We also reprogrammed MEGA to run natively on all major operating systems (Kumar *et al.* 2018) using a single codebase. On the way, we released a command-line version of MEGA for use in iterative and high-throughput analyses (Kumar *et al.* 2012). Today, the MEGA software family logs over 100,000 downloads annually by researchers and more than 300,000 downloads by students. These figures continue to astonish and inspire us. UItimately, these numbers reflect the central role of molecular evolutionary approaches in modern biological research, driven by genomics and computing advances (Kumar 2022).

As the analysis of sequence data sets became increasingly time- and memory-intensive, each new version of MEGA included innovations that enhance the computational efficiency of existing approaches and introduce new methods. These innovations address the bottlenecks encountered in molecular evolutionary analysis using both traditional and new approaches, not only on desktops but also on high-performance computing infrastructures. For example, we developed a relative rate framework that relaxes the molecular clock assumption for estimating divergence times, which has a solid theoretical foundation (Tamura *et al.* 2012, 2017). This approach, called RelTime, produces divergence time estimates orders of magnitude faster with minimal memory requirements compared to resource-intensive Bayesian approaches, yet RelTime maintains comparative accuracy. With this development, MEGA now provides extensive facilities for estimating divergence times and their confidence intervals, complementing the Bayesian approaches available in other software packages. More recently, we developed a phylogenomic subsampling and upsampling (PSU) framework, which serves as the basis for novel approaches to estimating bootstrap support values and selecting the optimal substitution models for large sequence alignments (Sharma and Kumar 2021, 2022). These approaches offer significant computational savings while generating results equivalent to those obtained by analyzing the entire data set. We are integrating PSU into the upcoming MEGA software releases.

## Equitable opportunities and sustainable science

The push for computational efficiency is not only a technical issue; it is also an ethical one. Scientists and students in areas with intermittent electricity or limited grant funding often have to prioritize which analyses they can afford to conduct. By reducing MEGA's memory and time footprint, we lower the barrier to participation and minimize the carbon footprint of computations (Kumar 2022). Essentially, greener algorithms enable a more inclusive scientific enterprise. Therefore, developing novel algorithms and methods that produce accurate results with minimal computational resources must be a key design imperative for all future approaches. They will democratize science, enable broad participation regardless of resource availability, and ensure that valuable data-driven discoveries are globally accessible and reproducible. These green computing principles will continue to guide MEGA's ongoing development and modernization, keeping pace with ever-growing sequence data sets (Kumar 2022).

## TimeTree: building a chronology of life

Data-driven discovery of species divergence times has been my passion since the early 1990s. In collaboration with S. Blair Hedges, I tested the hypothesis of adaptive radiation in mammalian and avian orders at the K-Pg boundary, coinciding with the extinction of the dinosaurs. Our estimates of species divergence times based on genes (molecular dates) placed many of these ordinal divergences much deeper in time, aligning with continental breakups rather than the dinosaur extinction (Hedges *et al.* 1996; Kumar and Hedges 1998). Molecular dates have often preceded times inferred from the fossil record for many other key events in the tree of life. The influx of new genomic data, the development of relaxed molecular clock methods, and varying interpretations and uses of the fossil record have led to numerous estimates of divergence times for many speciation events. Now, hundreds of studies are published annually that date species phylogenies, enhancing our knowledge of the molecular timescale of speciation (Kumar *et al.* 2022).

To make knowledge of molecular dates widely accessible, S. Blair Hedges and I initiated the development of a curated database of molecular dates, available online at www.timetree.org (Hedges *et al.* 2006). The intention was to provide a holistic and easily accessible view of the burgeoning literature on species divergence times. From the outset, TimeTree knowledge-base (TT-KB) was designed for broad utility, aiming to be equally useful to the public, scientists across disciplines, and taxonomic experts. It started with a simple, Google-like interface that allows users to query the divergence time between any 2 species using common or scientific names (Hedges *et al.* 2006). From its modest beginnings, incorporating published molecular dates and timetrees from 70 studies, the TimeTree database has grown to include molecular dates from over 4,000 studies (Kumar *et al.* 2022).

Developing and curating computation-friendly representations of thousands of timetrees published in these studies has led to numerous meta-analyses, including the construction of the largest timetree of life, which contains over 150,000 species, and the discovery of a clock-like pattern of speciation (Hedges *et al.* 2015; Kumar *et al.* 2022). The current TimeTree database can deliver a timetree for any user-specified subset of species and a timeline of organismal evolution for any given species (Kumar *et al.* 2022). Currently, over 250,000 TT-KB queries are launched each year by researchers, students, and the general public. A key principle driving TT-KB development has been to bridge the gap between specialized scientific research and broader public understanding. The use of common names alongside scientific classifications, coupled with a consistently user-friendly interface, allows anyone, regardless of their specific scientific background, to explore evolutionary relationships and learn about the deep history of life. This commitment to accessibility ensures that TimeTree serves not only as a research tool but also has a significant impact on science education and public engagement with evolutionary concepts. Overall, TT-KB addresses the need to make molecular dates, locked up in published literature, accessible and analyzable for studying evolutionary rates, biogeography, and the timing of major evolutionary events.

## Summary

In retrospect, MEGA's ongoing evolution from distance-based phylogeny reconstruction on resource-limited PCs to cross-platform, sophisticated analyses optimized for green computing exemplifies how methodological innovation can be coupled with deliberative software engineering to broaden participation and accelerate discovery. Complementarily, TimeTree knowledge-base consolidates diverse and disparate evolutionary inferences into a rigorously curated, computation-ready knowledge base that facilitates comparative analyses at unprecedented phylogenetic depth and scale. Together, these resources lower the technical and financial barriers to advanced evolutionary analysis; enable reproducible, large-scale phylogenomic and phylomedicine investigations; and foster a culture of open and sustainable science. Emerging developments, particularly the rise of artificial intelligence and machine learning in evolutionary analysis (Kumar and Sharma 2021, 2024; Allard and Kumar 2025; Allard *et al.* 2025), are poised to further illuminate the genetic tapestry of life and empower the next generation of data-driven explorations.
